# Effects of mental fatigue on technical performance in soccer players: A systematic review with a meta-analysis

**DOI:** 10.3389/fpubh.2022.922630

**Published:** 2022-07-22

**Authors:** He Sun, Kim Geok Soh, Alireza Mohammadi, Xuanji Wang, Zuchang Bin, Zijian Zhao

**Affiliations:** ^1^School of Physical Education Institute (Main Campus), Zhengzhou University, Zhengzhou, China; ^2^Department of Sport Studies, Faculty of Education Studies, Universiti Putra Malaysia, Selangor, Malaysia; ^3^Faculty of Business Management, City University Malaysia, Selangor, Malaysia; ^4^Faculty of Education, Beibu Gulf University, Qinzhou, China

**Keywords:** soccer, technical performance, athletic performance, motor skill, mental fatigue

## Abstract

**Background:**

Mental fatigue largely influences technical performance in soccer, including offensive and defensive skills. However, these effects on technical performance among the soccer players have not yet been aggregated to be assessed systematically.

**Objective:**

The purpose of the review was to evaluate the impact of mental fatigue on soccer players' overall technical skills.

**Methods:**

Drawing on Web of Science, PubMed, Scopus, and EBSCOhost (CENTRAL and SPORTDicus), an in-depth search was conducted. PICOS established the eligibility criteria to select the studies as follows: (i) population—healthy soccer players; (ii) intervention—involving any mental-fatigue-prompted protocol; (iii) comparison—control conditions (active or passive without inducing mental fatigue); (iv) outcomes—technical performance (offensive and defensive skill); and (v) study design—randomized controlled trials.

**Results:**

A total of eight studies were qualified for inclusion in the systematic literature review. Overall, the results indicate that mental fatigue had significant effects on technical skills, including offensive and defensive skills. Specifically, there were significant effects on errors (ES = 0.977; *p* < 0.001), number of tackles (ES = −0.739; *p* = 0.005), and the percentage of successful tackles (ES = −0.628; *p* = 0.022), while there were no significant effects on the number of passes (ES = 0.240; *p* = 0.328), the percentage of accurate passing (ES = −0.008; *p* = 0.985), and the number of successful passes (ES = −0.322; *p* = 0.217).

**Conclusion:**

Overall, a significant effect of mental fatigue on the technical performance (e.g., tackles and errors) of soccer players was detected, while no significant effects on passing skills were detected. Future studies may consider investigating technical performance together with other important results (e.g., decision-making skills or internal load).

**Systematic Review Registration:**

https://inplasy.com/inplasy-2022-2-0008/, Inplasy protocol 202220008.

## Introduction

Technical performance in sports refers to the capacity to effectively engage at a high standard (1). This is also referred to as skilled sports execution (2). For example, in soccer, the phrase defines how effectively players handle a soccer ball by effective disposals (e.g., shooting, dribbling, and passing) and tackling for the overall benefit of the entire team (3). Therefore, technical performance is crucial among the soccer players, and can even determine the outcome of competitions (4). However, the decrease in technical performance is associated with mental fatigue (5).

Generally recognized as a complex psychophysiological phenomenon, mental fatigue is a condition of fatigue caused by an increase in the demand for cognitive activities (6). Because of great cognitive effort, mental fatigue can be promoted by the neuro-modulation that comes with adenosine (7). With the increase in adenosine and the perception of effort, dopamine, and motivation both decrease, and this results in a decrease in the overall performance of the player (7). Generally, mental fatigue decreases one's ability to pay attention (8), reduces the reaction time (9, 10), and reduces motor skills (11). Particularly, mental fatigue influences soccer players more than in other sports (5), due to soccer competitions being practically longer than other sports and highly requiring cognitive activity.

Specifically, a variety of performances have been investigated and showed impairment among the soccer players, such as intermittent endurance (12, 13) and decision-making skills (14, 15). As for technical performance, mental fatigue can also reduce passing accuracy (16) and increase the number of poor passes (17). However, some studies have shown inconsistent results. For example, Ciocca et al. (18) found that mental fatigue did not influence the number of successful tackles. In addition, Smith et al. (19) did not find any influence on the reaction time of passing. Thus, it is difficult to establish the true effect among the soccer players.

Most recent studies have discussed the impact of mental fatigue on soccer players (5, 20, 21). Among them, only Grgic et al. evaluated technical performance with a meta-analysis. However, technical performance was not examined comprehensively. The authors only evaluated passing and shooting techniques using the Loughborough Soccer Task. Perhaps, more importantly, no one has performed a meta-analysis that gathered prominent data from previous studies that investigated technical performance regarding offensive and defensive skills in soccer. Fernandez-Navarro et al. (22) indicated that soccer players have different styles of play based on the role they play in their team (defensive or offensive). In addition, the needed technical performance differs based on their role, and research that disregards the difference among the various positions and roles of players on a team can result in inconclusive or biased findings and methodological issues (1).

As a result, this review presents a meta-analysis investigating the effect of mental fatigue on the overall technical performance of soccer players, which can assist in determining the optimal manner to manage this effect, in turn increasing overall performance during competitions or training. The study hypothesizes that mental fatigue greatly impacts soccer players' technical performance.

## Methods

**Figure 1:**
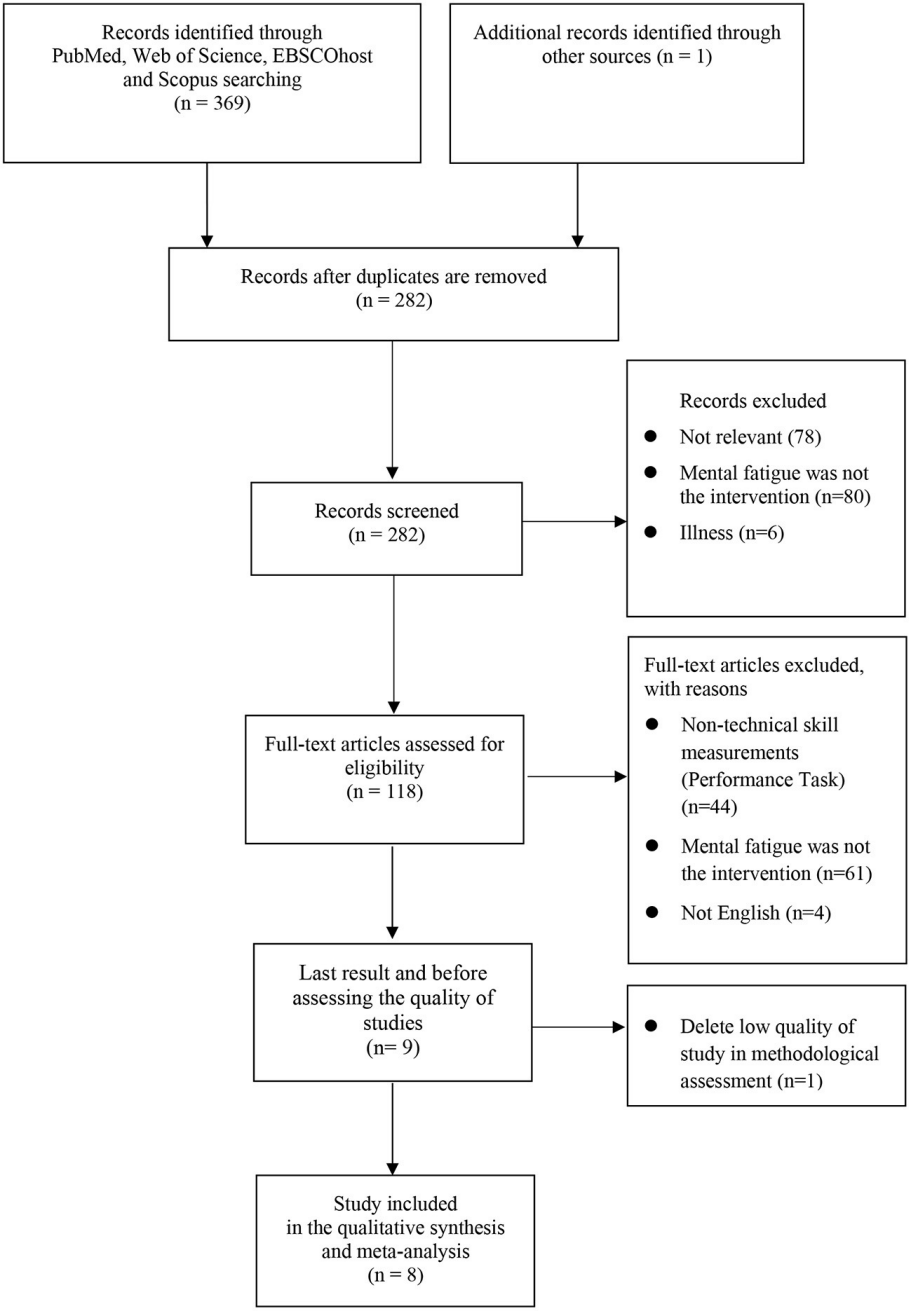
PRISMA selection process summary.

This review used the PRISMA protocol's list of preferred reporting items (23). A systematic search of the literature was conducted on four primary academic indexing databases, namely, Web of Science, PubMed, EBSCOhost (CENTRAL, Psychology and Behavioral Sciences Collection, and SPORTDicus), and Scopus, from their date of publication to March 2022. EBSCOhost comprises several sub-databases. However, only two were selected, namely, CENTRAL and SPORTDicus, primarily because their contents were more relevant. In addition, citations and reference lists were looked through to see if there were additional studies. The specifics of the search results are shown in Figure 1. Experienced librarians were assisted in the search to ensure that the searching method was carried out optimally.

In addition, the planned analyses considered in this study were performed on INPLASY (ref. 202220008). Even though INPLASY comprises protocols that investigate the effect of mental fatigue, they do not consider the overall technical performance of soccer players. Therefore, the proposed protocol is considered novel.

### Eligibility criteria

The PICOS approach was used to select the relevant studies (Table 1). The chosen studies had to be in English and published in academic journals. Studies were considered qualifying if the results were discussed in terms of the technical performance of soccer players. Cognitive tasks had to be employed to prompt mental fatigue before tests for the main outcome, with several kinds of cognitive events, and the participants merely comprised skilled soccer players. They were categorized into multiple levels. Moreover, the studies had to comprise a control group that had no mental fatigue and did not engage in mentally fatiguing tasks.

**Table 1:** Eligibility criteria based on PICOS (participation, intervention, comparison, outcome, and study design).

**PICOS**	**Criteria**
Participation	Soccer players
Intervention	fatigue induced by cognitive tasks
Comparison	Mentally fatigued vs. non-mentally fatigued players
Outcome	Technique
Study design	Randomized controlled trial

### Selection of studies

Keywords and Boolean operators were considered separately and in aggregation during the searching process involving the 5 above-mentioned databases (Supplementary Table 1). Specifically, the search strategy employed the following terms and operators: (“mental fatigue” OR “mental exertion” OR “cognitive fatigue” OR “cognitive exertion” OR “mental exhaustion” OR “mental tiredness”) AND (“athletic performance” OR “technical skill^*^” OR “skill^*^” OR “technique” OR “performance”) AND (soccer OR football). Moreover, the search was also thoroughly carried out on Google Scholar and references. The PICOS approach was employed to check if the studies were eligible to be included.

The title and abstract of each study were checked. Next, the full-text version of the articles was checked. Two independent reviewers performed this procedure. Any disagreement was further discussed. A third reviewer assisted until a consensus was reached if needed. Figure 1 depicts the selection procedure.

### Data extraction

The data that were taken from the literature included (i) authors and year of publication; (ii) characteristics of study participants (e.g., gender, training status); (iii) a description of the control and cognitive tasks; (iv) type of technical performance; (v) assessment of technical performance; and (vi) mean and standard deviation of the results for control and intervention groups. All of the information was then put into an Excel sheet.

### Quality assessment and risk of bias

Methodological quality was examined with the quantitative assessment instrument referred to as “QualSyst” (24), which comprises a total of 14 items (see Table 2). The scoring procedure depends on the extent to which a particular criterion is satisfied (no is 0, partial is 1, yes is 2). The final summary value of every study was computed. The calculation was carried out by two independent reviewers. A third senior reviewer was also requested to give an opinion for obtaining a reasonable consensus. Scores of ≤ 55%, 55–75%, and ≥75 indicated low, medium, and high quality, respectively.

**Table 2:** “Qualsyst” of quality assessment.

**Publication**	**Question/objective described**	**Appropriate study design**	**Appropriate subject selection**	**Characteristic sufficiently described**	**Random allocation**	**Researchers blinded**	**Subjects blinded**	**Outcome measures well-defined and robust to bias**	**Appropriate sample size**	**Analytic methods well-described**	**Estimate of variance reported**	**Controlled for confounding**	**Results reported in detail**	**Conclusion supported by results?**	**Rating**
Badin et al. (16)	1	2	2	1	NA	1	0	2	1	2	2	1	1	2	Medium
Smith et al. (12)	2	2	2	2	NA	0	1	2	1	2	2	1	2	2	High
Greco et al. (25)	1	1	1	2	1	0	0	1	0	2	1	0	1	1	Low
Smith et al. (19)	2	2	2	2	NA	2	1	2	1	2	2	1	2	2	High
Trecroci et al. (17)	2	2	2	2	NA	0	0	2	1	2	2	0	2	2	Medium
Filipas et al. (26)	2	2	2	2	NA	2	0	1	2	2	0	1	1	1	Medium
Soylu and Arslan (27)	2	2	1	2	NA	0	0	2	1	2	2	0	2	2	Medium
Ciocca et al. (18)	2	2	1	2	2	0	0	2	1	2	2	0	2	2	Medium
Soylu et al. (28)	2	2	1	2	NA	0	0	2	1	2	2	0	2	2	Medium

**Figure 2:**
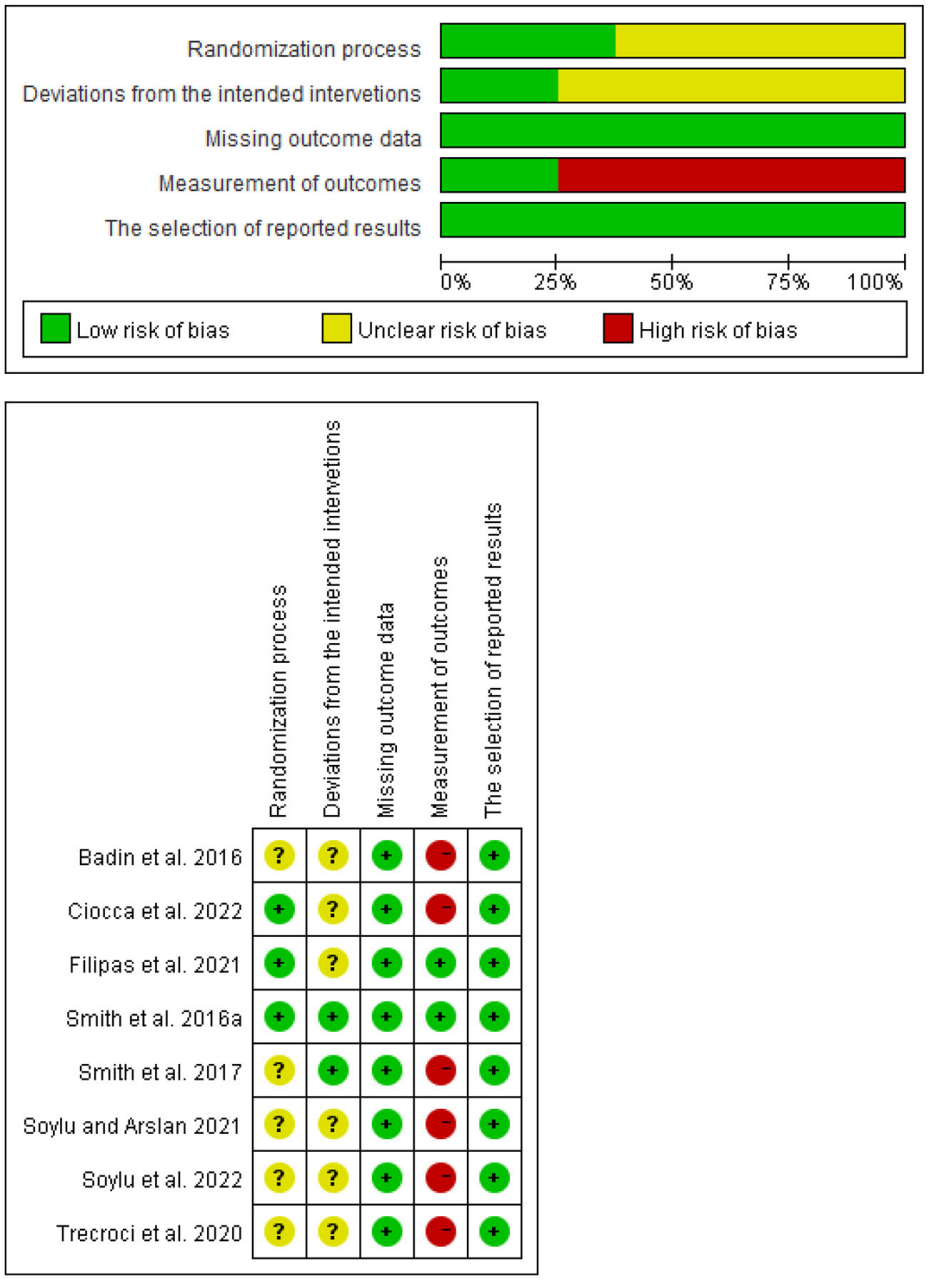
Risk of bias assessment using Rob 2 for included studies.

Following the Cochrane Collaboration guidelines, the risk of bias was assessed with RoB 2.0 (29). The signaling questions assigned a rating of “low risk of bias,” “some concerns of bias,” or “high risk of bias,” to each of the five domains (Figure 2). The overall risk of bias in each study was then determined.

Overall, two independent reviewers applied the QualSyst and RoB 2 tools, respectively. Either consensus was reached or a third reviewer settled disagreements.

### Statistical analysis

The mean and standard deviation (SD) values for the outcomes were considered to compute the effect sizes (ES; Hedge's g) for every result under two conditions (mental fatigue vs. control condition). In the absence of mean and SD, 95% confidence interval (CI) and standard error of the mean were used. The discussion of effect sizes relied on the following thresholds: trivial (or <0.20), small (or 0.20–0.6), moderate (or >0.6–1.2), large (or >1.2–2.0), very large (or >2.0–4.0), and extremely large (or >0.4) (30). A minus value showed a decrement in the outcomes. The random-effects model was used to carry out the meta-analysis (31). The *I*^2^ statistic was employed to assess the heterogeneity. *I*^2^ values were considered having low (or <50%), moderate (or 50–75%), or high heterogeneity (or >75%) (32). Egger's test determined whether bias risk existed (33). The threshold of statistical significance was defined to be *p* < 0.05. All of the analyses were carried out using the Comprehensive Meta-Analysis software (Biostat Inc., Englewood, NJ, USA; version 2).

## Results

### Study inclusion

This study found 369 unique publications. A total of nine studies were eligible after screening. Google Scholar and reference yielded one additional study. Two reviewers agreed on the outcome. The steps involved in selecting the studies are depicted in Figure 1.

### Quality assessment and risk of bias

Table 2 shows the overall methodological quality assessment with the tool of QualSyst. Notably, there was a study [see Table 2; (25)] that was of low quality and was not considered (24). Therefore, this review was based on eight research studies that analyzed soccer players' technical performance.

Regarding the risk of bias, the Rob 2 tool revealed that six studies (16–19, 27, 28) posed a high risk of bias, whereas the rest showed either unclear or low risk (Figure 2). The high risk of bias is relative to the domain of the measurement of the outcomes, according to the signaling questions. Specifically, there was no evidence that the outcome assessors were unaware of the intervention received in these six studies.

Also, five other studies (16, 17, 19, 27, 28) were categorized to have “unclear risk of bias” in the domain of the randomization process because allocation concealment was unknown. Finally, only Smith et al. (12) and Smith et al. (19) showed that participants and personnel were not aware of the intervention.

### Overview of studies

Table 3 shows the summary of the extracted studies. All the investigations on technical performance focused on offensive and defensive skills. Moreover, all studies used 30 min-Stroop events to prompt mental fatigue conditions, beside, one investigation used a 30 min-tactical task (18). Moreover, the analyzed studies were comprehensively examined from the lab (e.g., the Loughborough soccer pass assessment) to the field. Small-sided games (SSGs) were the primary tools to study the effect of mental fatigue on the technique of soccer players. However, the format varied. Trecroci et al. (17) used 4 vs. 4 with one wildcard player, while others mainly used 2 vs. 2 (27, 28), 3 vs. 3 (27, 28), 4 vs. 4 (27, 28) and 5 vs. 5 (16, 18) SSG formats without a wildcard. In addition, there were different SSG pitch dimensions in all investigations (Table 3).

**Table 3:** Overview of included studies.

**Publications**	**Subjects**	***N***	**Competitive level**	**Manipulation**	**Technique test**	**SSG format**	**Pitch dimension (m)**	**Technical outcome extracted**	**Technique type**
Badin et al. (16)	Elite	20	U18	30 min-Stroop	Field SSG	5 vs. 5	20 × 30	Pass (n)	Offensive
								Pass acc (%)	
								Errors (n)	
								Tackles (n)	Defensive
								Tackles suc (%)	
Smith et al. (12) Exp 2	Well-trained	14	UA	30 min-Stroop	Lab LSST			Shot Speed	Offensive
								Passing time (s)	
Smith et al. (19)	Well-trained	16	UA	30 min-Stroop	Lab LSPT			Passing time (s)	Offensive
								Suc pass (n)	
								Errors (n)	
Trecroci et al. (17)	Sub-elite	10	U19	30 min-Stroop	Field SSG	4 vs. 4 + 1 w	32 × 40	Pass (n)	Offensive
								Pass acc (%)	
								Errors (n)	
								Tackles (n)	Defensive
								Tackles suc (%)	
Filipas et al. (26)	Elite	12	U14	30 min-Stroop	Lab LSPT			Passing time (s)	Offensive
								Shot Speed (*km*/*h*^−1^)	
		12	U16					Passing time (s)	
								Shot Speed (*km*/*h*^−1^)	
		12	U18					Passing time (s)	
								Shot Speed (*km*/*h*^−1^)	
Soylu and Arslan (27)	Amateur	18	UA	30 min-Stroop	Field SSG	2 vs. 2	15 × 27	Error (n)	Offensive
								Tackles (n)	Defensive
								Tackles suc (%)	
						3 vs. 3	20 × 30	Error (n)	Offensive
								Tackles (n)	Defensive
								Tackles suc (%)	
						4 vs. 4	25 × 32	Error (n)	Offensive
								Tackles (n)	Defensive
								Tackles suc (%)	
Ciocca et al. (18)	Elite	10	U18	30 min-tactical tasks	Field SSG	5 vs. 5	26 × 36	Pass (n)	Offensive
								Suc Pass (n)	
								Pass acc (%)	
								Error (n)	
								Tackles (n)	Defensive
								Tackles suc (%)	
Soylu et al. (28)	Sub-elite	24	U16	30 min-Stroop	Field SSG	2 vs. 2	15 × 27	Suc Pass (n)	Offensive
								Error (n)	
								Tackle (n)	Defensive
						3 vs. 3	20 × 30	Suc Pass (n)	Offensive
								Error (n)	
								Tackle (n)	Defensive
						4 vs. 4	25 × 32	Suc Pass (n)	Offensive
								Error (n)	
								Tackle (n)	Defensive

*acc, accuracy; suc, success*.

Notably, even though two of the studies could be considered for the meta-analysis discussion (34), small sample sizes are typical in the literature on sports science (35), and the interpretation of the results obtained in this review and meta-analysis was only carried out if three or more study groups were considered for the outcome data for this metric. A total of six meta-analysis studies are considered in the below sections, for (i) the number of passes (16–18); (ii) the percentage of accurate passe (16–18, 27); (iii) the number of successful passes (18, 19, 28); (iv) the number of errors (16–19, 27, 28); (v) the number of tackles (16–18, 27, 28); (vi) the percentage of successful tackles (16–18, 27). Therefore, two studies (12, 26) were only included in the review without meta-analysis, as the number of investigations did not reach three.

### The effect of mental fatigue on passes

The effect of mental fatigue on passes was mainly investigated by analyzing the number of passes, the percentage of accurate passes, and the number of successful passes (Table 3). These three aspects are examined in the following sections.

#### The effect of mental fatigue on the number of passes

**Figure 3:**
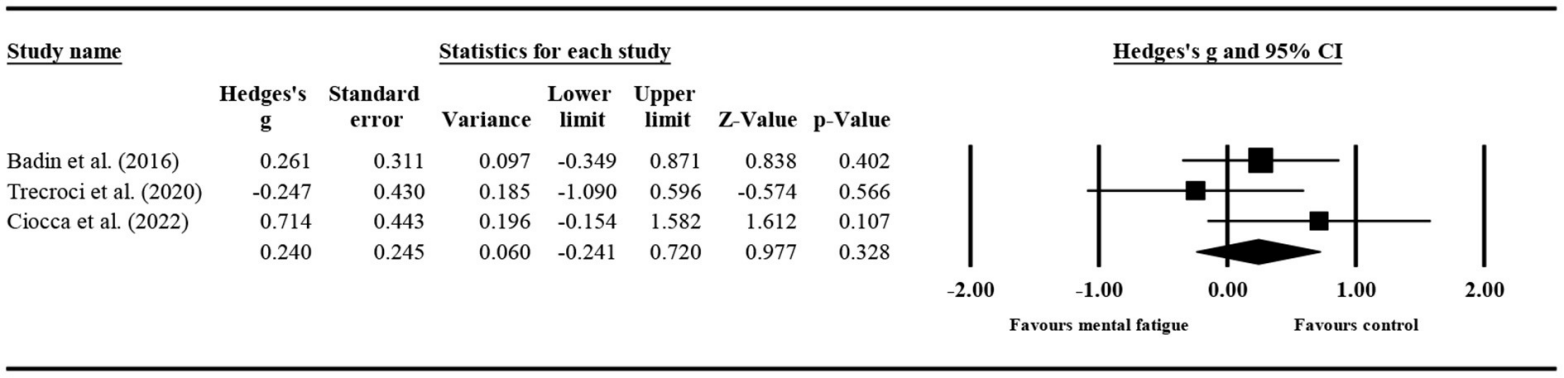
Forest plot graph of deviation in the number of passes with the comparison of two conditions (mental fatigue and control condition). The results displayed are effect sizes with 95% CIs.

Three studies (Figure 3) provided data on the number of passes, involving three mental fatigue and three control conditions (pooled *n* = 80). No significant impact of mental fatigue on the number of passes (ES = 0.240; 95% CI = from −0.241 to 0.720; *p* = 0.328; Egger's test *p* = 0.496) was detected. Moreover, there was no significant heterogeneity for the overall effect (τ^2^ = 0.033; Q = 2.433; *P* = 0.296; *I*^2^ = 17.791%). The weight value of every study was in the range of 26.210 to 46.220%.

#### The effect of mental fatigue on the percentage of accurate passing

**Figure 4:**
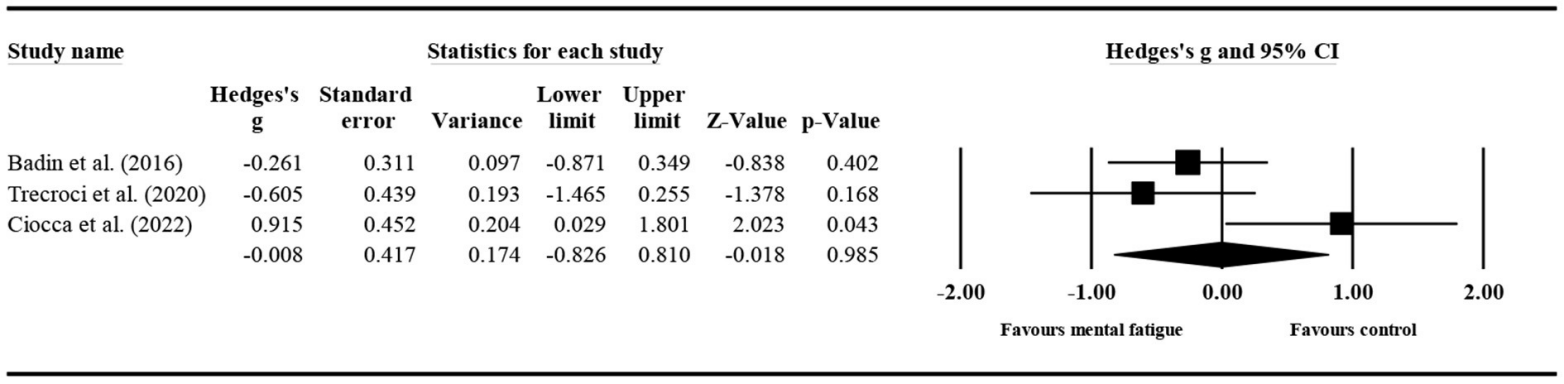
Forest plot of changes in the percentage of accurate passing with the comparison of two conditions (mental fatigue and control condition). The results displayed are effect sizes with 95% CIs.

Three studies (Figure 4) provided data on the percentage of accurate passing, involving three mental fatigue and three control conditions (pooled *n* = 80). Mental fatigue had no significant effects on the percentage of accurate passing (ES = −0.008; 95% CI = from −0.826 to 0.810; *p* = 0.985; Egger's test *p* = 0.366). Moreover, there was a moderate heterogeneity for the overall effect (τ^2^ = 0.363; Q = 6.604; *P* = 0.037; *I*^2^ = 69.713%). The weight value of every study ranged from 30.720 to 37.900% in the analysis.

#### The effect of mental fatigue on the number of successful passes

**Figure 5:**
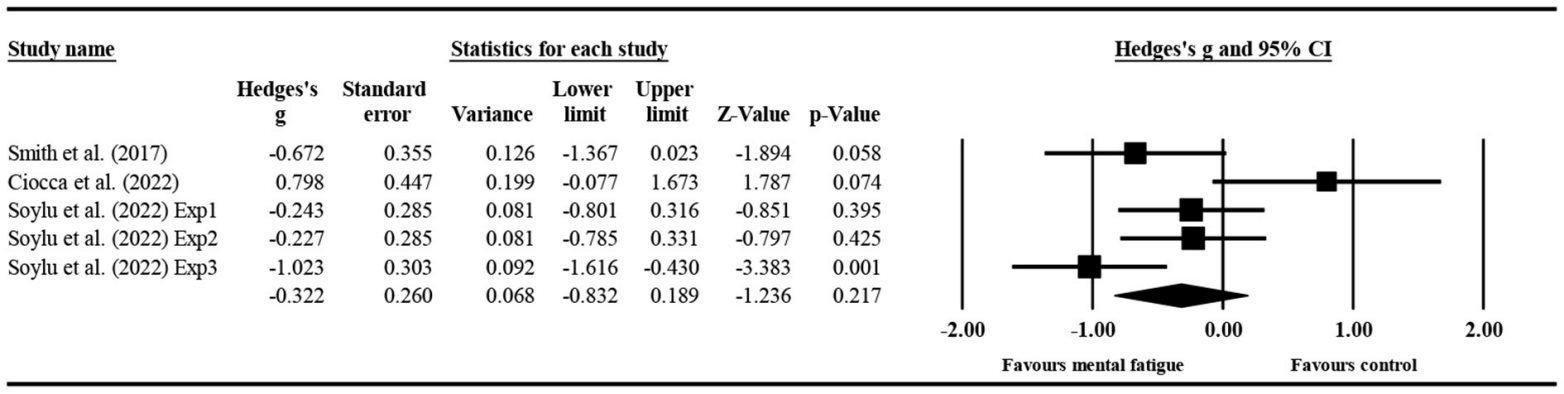
Forest plot of changes in the number of successful passes with the comparison of two conditions (mental fatigue and control condition). The results displayed are effect sizes with 95% CIs.

Three studies (Figure 5) provided data on the successful passes, involving five mental fatigue and five control conditions (pooled *n* = 196). Mental fatigue had no significant effects on the successful passes (ES = −0.322; 95% CI = from −0.832 to 0.189; *p* = 0.217; Egger's test *p* = 0.178). Moreover, there was a moderate heterogeneity for the overall effect (τ^2^ = 0.229; Q = 12.681; *P* = 0.013; *I*^2^ = 68.457%). The weight value of every study in the analysis ranged from 15.850 to 21.910%.

#### The effect of mental fatigue on errors

**Figure 6:**
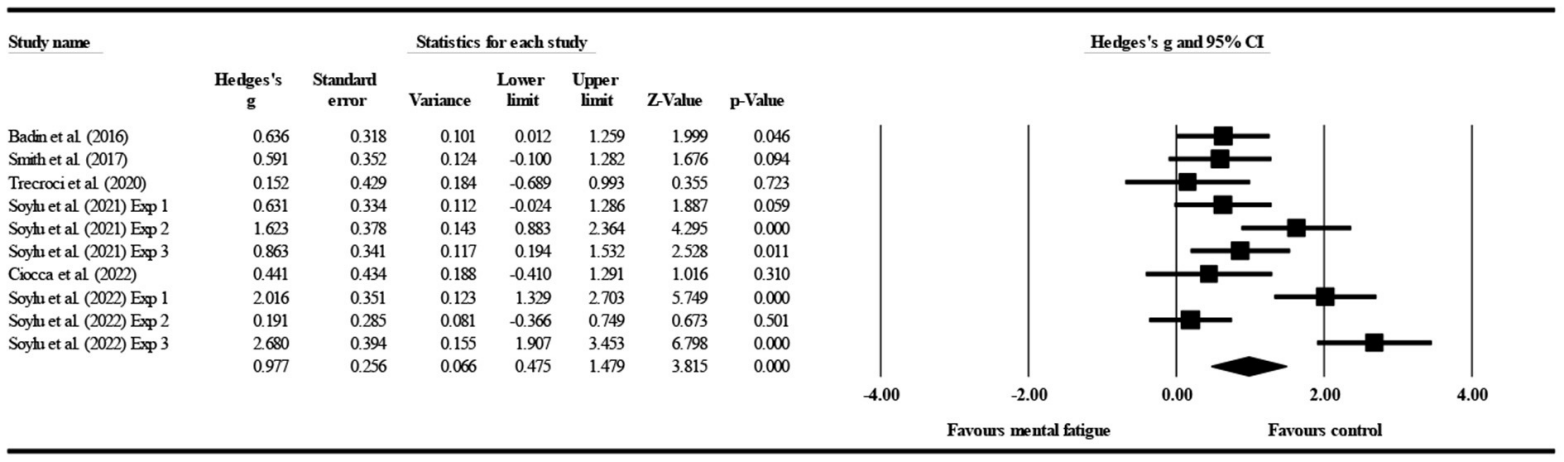
Forest plot of changes in errors with the comparison of two conditions (mental fatigue and control condition). The results displayed are effect sizes with 95% CIs.

Six studies (Figure 6) provided data on errors, involving ten mental fatigue and 10 control conditions (pooled *n* = 364). Mental fatigue had a significant effect on errors (ES = 0.977; 95% CI = from 0.475 to 1.479; *p* < 0.001; Egger's test *p* = 0.197). Moreover, there was a high heterogeneity for the overall effect (τ^2^ = 0.525; Q = 46.580; *P* < 0.001; *I*^2^ = 80.678%). The weight value of every study ranged from 9.200 to 10.830% in the analysis.

### The effect of mental fatigue on tackles

#### The effect of mental fatigue on the total number of tackles

**Figure 7:**
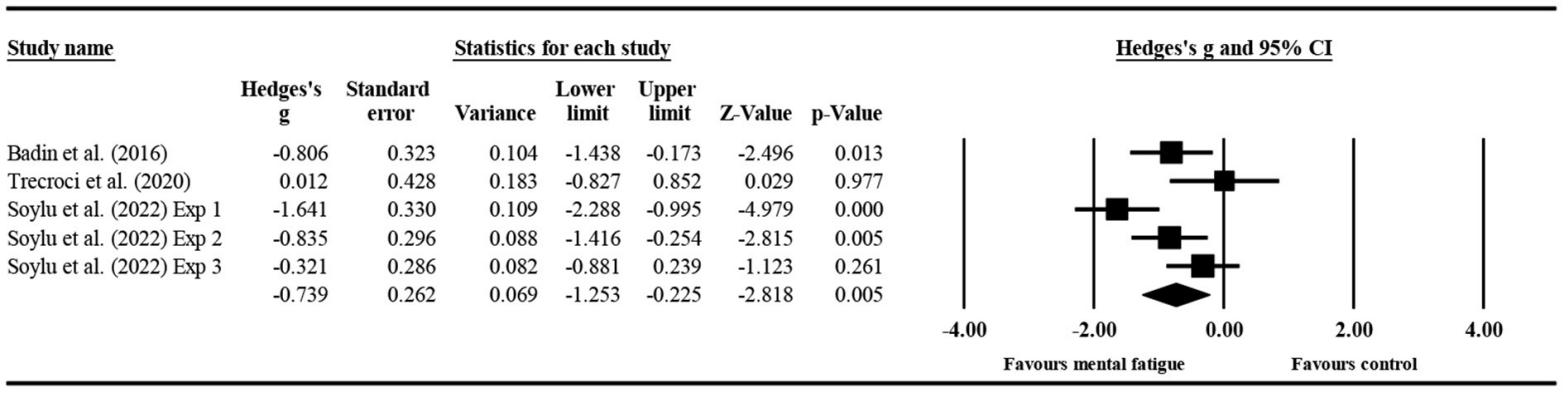
Forest plot of changes in the total number of tackles with the comparison of two conditions (mental fatigue and control condition). The results displayed are effect sizes with 95% CIs.

Three studies (Figure 7) provided data for the total number of tackles, involving five mental fatigue and five control conditions (pooled *n* = 204). Mental fatigue had a significant effect on the total number of tackles (ES = −0.739; 95% CI = from −1.253 to −0.225; *p* = 0.005; Egger's test *p* = 0.372). Moreover, there was a moderate heterogeneity for the overall effect (τ^2^ = 0.234; Q = 12.841; *P* = 0.012; *I*^2^ = 68.850%). The weight value of every study ranged from 16.470 to 21.780% in the analysis.

#### The effect of mental fatigue on the percentage of successful tackles

**Figure 8:**
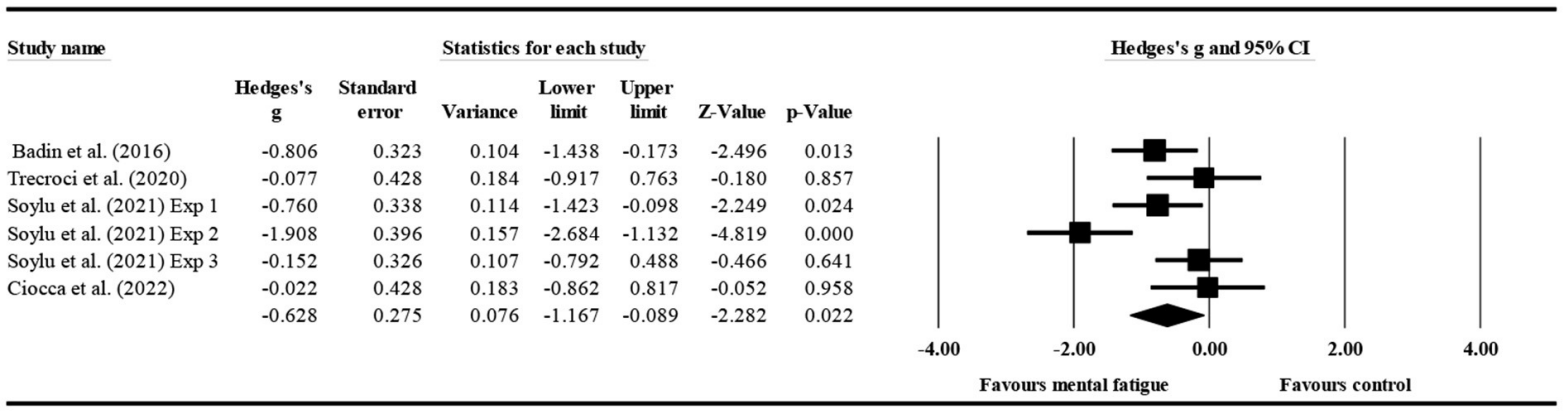
Forest plot of changes in the percentage of successful tackles with the comparison of two conditions (mental fatigue and control condition). The results displayed are effect sizes with 95% CIs.

Four studies (Figure 8) provided data for the percentage of successful tackles, involving five mental fatigue and five control conditions (pooled *n* = 188). Mental fatigue had a significant effect on the percentage of successful tackles (ES = −0.628; 95% CI = from −1.167 to −0.089; *p* = 0.022; Egger's test *p* = 0.452). Moreover, there was a moderate heterogeneity for the overall effect (τ^2^ = 0.315; Q = 16.684; *P* = 0.005; *I*^2^ = 70.032%). The weight value of every study ranged from 15.170 to 18.050% in the analysis.

## Discussion

This review analyzes the effect of mental fatigue on the overall technique of soccer players, including offensive and defensive performance. The main results reveal that mental fatigue negatively influences defensive (e.g., tackle) and offensive techniques (e.g., error) (Technique type: Table 3). However, the evidence reported in the studies does not support that mental fatigue adversely affects passing skills (e.g., 19, 31).

### Effect of mental fatigue on offensive technique

As an important measurement of offensive techniques, passing determines the successful performance of soccer players. Previous investigations have demonstrated that successful teams can complete more passes (36) and that a significant percentage of goals is achieved by passes (37). However, this is significantly impaired by mental fatigue, as reported in a prior study (12, 19). Inconsistently, this review could not provide evidence to support this conclusion from the meta-analysis and detected that there were no major differences in the number of passing, the percentage of accurate passes, and the number of successful passes between mental fatigue and control conditions. Since two prestigious investigations by Smith and his colleagues were performed in a lab setting, it prompts several questions. For instance, does the impairment of mental fatigue on passing performance in a lab setting truly occur in actual games? Perhaps, more likely, players that perform in ecological settings (e.g., SSG: Table 3) could be more freely paced. Despite the best efforts to standardize them, some factors, which might be adjusted to compensate for mental fatigue, still cannot be closely controlled, such as the action of opponents, and the trajectory paths of the ball.

In addition, it should be considered that the number of passes, the percentage of accurate passing, and the number of successful passes may not be the optimal metrics for assessing mental fatigue. Several investigations have demonstrated that mental fatigue negatively influences decision-making skills in soccer players (14, 15, 38). That is, mentally fatigued players might put in less effort and select a more conservative approach. Thus, simply measuring the number of passes or successful passing might not be accurate or enough for capturing mental fatigue in SSGs.

Notably, mental fatigue activates the anterior cingulate cortex (ACC) in the frontal part, possibly leading to a rise in adenosine and reducing the concentration (39, 40). These changes in the ACC are responsible for the impairment of executive functions such as attention (8, 41) and performance adjustment (40). Thus, comprehensive evidence has demonstrated that mental fatigue hinders motor skills (11, 42). Therefore, a significant difference in errors was detected in the current meta-analysis. However, data from the included articles showed that the heterogeneity was high—this should be investigated in future studies.

### Effects of mental fatigue on defensive techniques

Since the performance of soccer players is impacted not only by offensive approaches but also by defensive ones, comprehending the effect of mental fatigue on defensive tackling could be valuable to soccer coaches. It could optimize a team's overall performance or game strategy, especially near the end of a game (7).

Previous studies have shown that cognitive resources are used for offensive skills more than for their defensive counterparts (43). Therefore, mental fatigue may have a greater impact on offensive skills than on defensive ones, since perception rises more significantly in the former. However, the current meta-analysis indicates that mental fatigue has a great impact on defensive techniques, for the reported tackle number and successful tackles were significantly higher. Therefore, this finding indicates that defensive skills have a greater likelihood to be impacted by mental fatigue. On the other hand, this must be empirically confirmed in future studies *via* investigations with a direct comparison between these two technical skills.

Notably, all the measures of defensive techniques were recorded during SSGs, which offer an effective and efficient training approach, as opposed to the conventional aerobic approach (44, 45). However, players' number (46), pitch dimensions (47, 48), and rule modifications (46, 47) could largely influence the intensity and cognitive demands. Therefore, could these intensity and cognitive demands influence different levels of mental fatigue, independently causing the impairment of technical skills? Although Soylu and Arslan (27) and Soylu et al. (28) conducted investigations and showed that 2 on 2, 3 on 3, and 4 on 4 influenced technical skills differently among the mentally fatigued soccer players, other SSG formats need to be examined. It is typical for coaches to adjust the rules of SSGs to modify the technical load on the players, for instance, by limiting the number of ball possessions per player or adding an offside limit, which is considered to replicate a game's technical demand (ball possession and passes).

### Study limitations and recommendations for future studies

This review has noteworthy limitations. First, we only focused on overall performance, which is defined as defensive or offensive skills, and excluded players' physical performance and decision-making skills. Since these outcomes contribute to the overall performance of sports players, it is suggested to examine them together to obtain a more comprehensive view in future studies. Second, only choosing studies in English may have limited the results.

The consideration of possible covariance was largely absent in the included studies, such as the trait of self-control, which has been recognized as an ability to manage finite cognitive resources (49, 50). The most recent study showed that these finite resources may have a counteractive effect on mental fatigue (51). Although mental fatigue is a “state” condition, it is crucial to control this “trait” for future investigation, because it might be a factor that influences the degrees of mental fatigue.

Moreover, mental-fatigue-promoting protocols can be compared against mental boosting strategies (such as music or exposure to nature). The difference between the two selections can provide valuable information for coaches to create effective training strategies. Nature exposure, for example, has been shown to mitigate performance declines caused by mental fatigue (52). As a result, soccer players can benefit from the visualization or guided imagery training using natural stimuli. This could be a tool that players can use in many situations to reduce mental fatigue (e.g., training or competition) as a resource and technique to activate on demand.

Music listening was reported to be largely accepted by about 82% of players during the period before a match (53). Moreover, motivational music was shown to improve the force output in soccer players in comparison with conditions without music (54).

## Conclusion

This review shows that mental fatigue has a great impact on the offensive and defensive skills of soccer players. However, it is vital to discuss the results carefully, since other important results (e.g., decision-making skills or internal load) have been extensively investigated. The effect of mental fatigue considering factors such as various ages, SSG formats, and pitch dimensions may have a moderating impact; thus, it should be considered in future studies.

## Data availability statement

The datasets generated during and/or analyzed during the current study are available from the corresponding author upon reasonable request.

## Author contributions

All authors participated in the documentation, development, and writing of the manuscript. This study was reviewed by all authors and all of them were responsible for its contents and the final version.

## Funding

This research was funded by the National Social Science Fund of China (Grant No. 21ATY009).

## Conflict of interest

The authors declare that the research was conducted in the absence of any commercial or financial relationships that could be construed as a potential conflict of interest.

## Publisher's note

All claims expressed in this article are solely those of the authors and do not necessarily represent those of their affiliated organizations, or those of the publisher, the editors and the reviewers. Any product that may be evaluated in this article, or claim that may be made by its manufacturer, is not guaranteed or endorsed by the publisher.
