# Supplementary material for: Effects of mental fatigue on technical performance in soccer players: A systematic review with a meta-analysis
Source: Front Public Health. 2022 Jul 22;10:922630. doi: 10.3389/fpubh.2022.922630 (PMC9354787; doi:10.3389/fpubh.2022.922630)
Supplement: Supplementary file 1 [file Table_1.DOC]

**Table S1 - Detailed search strategy**

| **Database** | **Search Keywords** | **Results** |
| --- | --- | --- |
| Pubmed | 1. (("mental fatigue" OR "mental exertion" OR "cognitive fatigue" OR "cognitive exertion" OR "mental exhaustion" OR "mental tiredness"), **3,273** 2. ("athletic performance" OR "technical skill*" OR "skill*" OR "technique" OR "performance")), [**2,549,922**](https://pubmed.ncbi.nlm.nih.gov/?term=%22athletic+performance%22+OR+%22technical+skill%2A%22+OR+%22skill%2A%22+OR+%22technique%22+OR+%22performance%22&ac=no&sort=relevance) 3. (soccer OR football), [**24,710**](https://pubmed.ncbi.nlm.nih.gov/?term=%22soccer%22+OR+%22football%22&ac=no&sort=relevance) 4. (1) AND (2) AND (3), **41** | 41 |
| Web of Science | 1. Ts = (“mental fatigue” OR “mental exertion” OR “cognitive fatigue” OR “cognitive exertion” OR “mental exhaustion” OR “mental tiredness”), **6,392** 2. Ts = (“athletic performance” OR “technical skill*” OR “skill*” OR “technique” OR “performance”), [**15,209,340**](https://www.webofscience.com/wos/alldb/summary/c460be5a-ee53-4a40-a5be-df3211739503-2cd2008e/relevance/1) 3. Ts = (soccer OR football), [**94,575**](https://www.webofscience.com/wos/alldb/summary/a9257e2c-6a9d-4498-9735-0cb2cc9f04c4-2cd214cd/relevance/1) 4. (1) AND (2) AND (3), **85** | 85 |
| EBSCOhost | 1. “mental fatigue” OR “mental exertion” OR “cognitive fatigue” OR “cognitive exertion” OR “mental exhaustion” OR “mental tiredness”, **732** 2. "athletic performance" OR "technical skill*" OR "skill*" OR "technique" OR "performance", **97,881** 3. soccer OR football, **18,878** 4. (1) AND (2) AND (3), **85** | 192 |
| Scopus | 1. TITLE-ABS-KEY ( ( "mental fatigue"  OR  "mental exertion"  OR  "cognitive fatigue"  OR  "cognitive exertion"  OR  "mental exhaustion"  OR  "mental tiredness" )  AND  ( "athletic performance"  OR  "technical skill*"  OR  "skill*"  OR  "technique"  OR  "performance" ) AND  ( soccer  OR  football ) ), **51** | 51 |
